# Supplementary material for: Complexome profiling of the Chlamydomonas psb28 mutant reveals TEF5 as an early PSII assembly factor
Source: Plant Cell. 2025 Mar 17;37(6):koaf055. doi: 10.1093/plcell/koaf055 (PMC12164590; doi:10.1093/plcell/koaf055)
Supplement: koaf055_Supplementary_Data [file koaf055_supplementary_data.zip › psb28_interactive.zip!/plots/intensity/qualityPlots/peptides/CYP771A1_Cre08.g384000.t1.1.html]

### Cre08.g384000.t1.1

Trivial name: CYP771A1  
SeqCoverage [median]: 7.30  
Mass: 179.05 kDa  
  
 

| Sequence | MissedCleavage | Mass [Da] | wtIntensity | muIntensity | Score |
| --- | --- | --- | --- | --- | --- |
| AAAAQAAAAAAR | 0 | 1012.54 | 845.06 | 1643.51 | 181.55 |
| AAAASAAAAAAR | 0 | 971.51 | 1156.12 | 2738.59 | 200.07 |
| AGAGAAGVAK | 0 | 771.42 | 0.00 | 731.10 | 131.04 |
| DALAPAFRPDAVR | 1 | 1397.74 | 0.00 | 4207.25 | 45.91 |
| EVLGVLGGR | 0 | 898.52 | 220.74 | 610.86 | 62.72 |
| KGEPPLAAAIAAAR | 1 | 1334.77 | 0.00 | 716.33 | 36.45 |
| QAAADAAAAVGK | 0 | 1042.54 | 262.81 | 0.00 | 58.25 |
| TLASAASAR | 0 | 846.46 | 763.31 | 521.93 | 62.55 |
| VSAFLGAGLATQPDR | 0 | 1501.79 | 0.00 | 640.22 | 72.94 |
| VSAFLGAGLATQPDRQR | 1 | 1785.95 | 0.00 | 547.89 | 37.58 |
|  |  |  | 3248.0 | 12357.7 |  |
